# Randomized Trial of Complete Versus Lesion-Only Revascularization in Patients Undergoing Primary Percutaneous Coronary Intervention for STEMI and Multivessel Disease: The CvLPRIT Trial

**DOI:** 10.1016/j.jacc.2014.12.038

**Published:** 2015-03-17

**Authors:** Anthony H. Gershlick, Jamal Nasir Khan, Damian J. Kelly, John P. Greenwood, Thiagarajah Sasikaran, Nick Curzen, Daniel J. Blackman, Miles Dalby, Kathryn L. Fairbrother, Winston Banya, Duolao Wang, Marcus Flather, Simon L. Hetherington, Andrew D. Kelion, Suneel Talwar, Mark Gunning, Roger Hall, Howard Swanton, Gerry P. McCann

**Affiliations:** ∗ Department of Cardiovascular Sciences, University of Leicester and National Institute of Health Research Leicester Cardiovascular Biomedical Research Unit, Glenfield Hospital, Leicester, United Kingdom; † Department of Cardiology, Royal Derby Hospital, Derby, United Kingdom; ‡ Multidisciplinary Cardiovascular Research Centre and the Division of Cardiovascular and Diabetes Research, Leeds Institute of Cardiovascular and Metabolic Medicine, University of Leeds, Leeds, United Kingdom; § Department of Cardiology, Leeds Teaching Hospitals NHS Trust, Leeds, United Kingdom; ‖ Clinical Trials and Evaluation Unit, Royal Brompton & Harefield NHS Foundation Trust and Imperial College London, London, United Kingdom; ¶ University Hospital Southampton and Faculty of Medicine, University of Southampton, Southampton, United Kingdom; # Royal Brompton & Harefield NHS Trust, London, United Kingdom; ∗∗ University Hospitals of Leicester NHS Trust, Leicester, United Kingdom; †† National Institute for Health Research Cardiovascular Biomedical Research Unit, Royal Brompton & Harefield NHS Trust, London, United Kingdom; ‡‡ Department of Clinical Sciences, Liverpool School of Tropical Medicine, Liverpool, United Kingdom; §§ Norfolk and Norwich University Hospitals NHS Foundation Trust and Norwich Medical School, University of East Anglia Norwich, United Kingdom; ‖‖ Kettering General Hospital, Kettering, United Kingdom; ¶¶ Oxford Heart Centre, John Radcliffe Hospital, Oxford University Hospitals NHS Trust, Oxford, United Kingdom; ## Royal Bournemouth Hospital, Bournemouth, United Kingdom; ∗∗∗ Royal Stoke University Hospital, University Hospitals of North Midlands NHS Trust, Stoke-on-Trent, Staffordshire, United Kingdom; ††† The Heart Hospital, University College London Hospitals, London, United Kingdom

**Keywords:** complete revascularization, non-infarct-related lesion, primary percutaneous coronary angioplasty, FFR, fractional flow reserve, HF, heart failure, IRA, infarct-related artery, MACE, major adverse cardiac event(s), MI, myocardial infarction, MPS, myocardial perfusion scintigraphy, N-IRA, non–infarct-related artery, PCI, percutaneous coronary intervention, P-PCI, primary percutaneous coronary intervention, STEMI, ST-segment elevation myocardial infarction

## Abstract

**Background:**

The optimal management of patients found to have multivessel disease while undergoing primary percutaneous coronary intervention (P-PCI) for ST-segment elevation myocardial infarction is uncertain.

**Objectives:**

CvLPRIT (Complete versus Lesion-only Primary PCI trial) is a U.K. open-label randomized study comparing complete revascularization at index admission with treatment of the infarct-related artery (IRA) only.

**Methods:**

After they provided verbal assent and underwent coronary angiography, 296 patients in 7 U.K. centers were randomized through an interactive voice-response program to either in-hospital complete revascularization (n = 150) or IRA-only revascularization (n = 146). Complete revascularization was performed either at the time of P-PCI or before hospital discharge. Randomization was stratified by infarct location (anterior/nonanterior) and symptom onset (≤3 h or >3 h). The primary endpoint was a composite of all-cause death, recurrent myocardial infarction (MI), heart failure, and ischemia-driven revascularization within 12 months.

**Results:**

Patient groups were well matched for baseline clinical characteristics. The primary endpoint occurred in 10.0% of the complete revascularization group versus 21.2% in the IRA-only revascularization group (hazard ratio: 0.45; 95% confidence interval: 0.24 to 0.84; p = 0.009). A trend toward benefit was seen early after complete revascularization (p = 0.055 at 30 days). Although there was no significant reduction in death or MI, a nonsignificant reduction in all primary endpoint components was seen. There was no reduction in ischemic burden on myocardial perfusion scintigraphy or in the safety endpoints of major bleeding, contrast-induced nephropathy, or stroke between the groups.

**Conclusions:**

In patients presenting for P-PCI with multivessel disease, index admission complete revascularization significantly lowered the rate of the composite primary endpoint at 12 months compared with treating only the IRA. In such patients, inpatient total revascularization may be considered, but larger clinical trials are required to confirm this result and specifically address whether this strategy is associated with improved survival. (Complete Versus Lesion-only Primary PCI Pilot Study [CvLPRIT]; ISRCTN70913605)

Primary percutaneous coronary intervention (P-PCI) is the standard of care for patients with ST-segment elevation myocardial infarction (STEMI). In up to 30% of such patients, significant stenoses are seen in 1 or more non–infarct-related arteries (N-IRA) during index angiography 1, 2.

It remains unresolved whether complete revascularization should be undertaken in this setting, with historical data providing conflicting evidence on the benefit and safety of immediate complete revascularization versus delayed complete revascularization versus revascularization as clinically required. In registry series, delayed complete revascularization appears to confer benefit, whereas observational studies to date have generally suggested no benefit and possible harm from immediate complete revascularization 3, 4. The prevailing uncertainty regarding optimal management has persisted despite the recent PRAMI (Preventive Angioplasty in Myocardial Infarction) trial, which demonstrated benefit from complete revascularization during the index procedure (5).

CvLPRIT (Complete Versus Lesion-Only Primary PCI trial) is a U.K. multicenter, randomized, open-label trial, which set out to test the feasibility, safety, and potential benefit of undertaking in-hospital complete revascularization of angiographically significant N-IRA lesions in patients presenting with P-PCI for STEMI compared with percutaneous coronary intervention (PCI) of the infarct-related artery (IRA) alone. The hypothesis was that early treatment of significant N-IRA lesions during the index admission would reduce global ischemic burden and protect against short- and medium-term recurrent ischemic events. CvLPRIT and PRAMI ask a similar question but were initiated independently and with definitive differences in trial design.

## Methods

The study was initially conceived and funded as a pilot trial, with the first patient recruited in May 2011 at Glenfield Hospital, and then rolled out to 3 centers (Leeds General Infirmary, Southampton General Hospital, and Harefield Hospital) over the subsequent 6 months. With additional support from the funders, the study was extended to include 3 more sites (Kettering General Hospital, Royal Derby Hospital, and Royal Bournemouth Hospital). The sample size was determined after the publication of a study by Politi et al. (6). The study rationale, design, and power calculation were published previously (7). The last patient was randomized in May 2013, and 12-month follow-up was completed in May 2014. The trial was conducted according to the Declaration of Helsinki and approved by the Trent Research Ethics Committee (reference No. 11/H0405/4) and registered with International Standard Randomized Controlled Trial Number (ISRCTN) 70913605.

Patients were recruited from those presenting with STEMI at 7 U.K. interventional centers. After electrocardiographic confirmation of STEMI, contemporary oral antiplatelet agents were administered (aspirin 300 mg plus clopidogrel 600 mg loading dose followed by 75 mg maintenance, prasugrel 60 mg loading dose and 10 mg daily, or ticagrelor 180 mg loading dose and 90 mg twice daily). Patients presenting within 12 h of symptom onset were considered for the trial if they fulfilled the initial inclusion criteria, with no exclusions (Online Table 1). Potentially eligible patients were asked to provide Ethics Committee–approved verbal assent before coronary angiography, which was undertaken via the femoral or radial artery, according to operator preference. If patients fulfilled inclusion criteria after angiography, and before IRA P-PCI, randomization was undertaken via a 24-h automated, voice-activated central system. Patients were randomized to 1 of 2 groups after P-PCI: either complete revascularization (including all N-IRAs) or IRA-only treatment. Randomization was stratified by infarct location (anterior/nonanterior) and symptom onset (≤3 h or >3 h).

P-PCI was undertaken according to current guideline recommendations and operators’ routine practice and could include aspiration thrombectomy, heparin, bivalirudin, or glycoprotein IIb/IIIa inhibitor. To reduce risk of in-stent restenosis, unless clinically contraindicated, drug-eluting stents (DES) were recommended for both IRA and N-IRA lesions. If randomized to complete revascularization, it was mandated that the IRA be treated first. If there were no clinical contraindications, complete revascularization was recommended at the same sitting to reduce multiple vascular punctures, avoid prolonged hospitalization, and attenuate potential patient dropout. If the operator decided for clinical reasons that the procedure be staged, it was mandated that the N-IRA be treated during the index admission.

Patients were provided with full trial information within 24 h of PCI and were approached for written consent to continue in the study. Patients were treated after the procedure with contemporary optimal medical therapy. Cardiovascular magnetic resonance imaging was performed as a pre-specified substudy in 205 patients on 1.5-T scanners, as described previously (8), at a median of 2.9 days (interquartile range [IQR]: 2.0 to 3.9 days) after P-PCI. Blinded cardiac magnetic resonance analysis was undertaken at the University of Leicester core laboratory, and the results will be analyzed and reported separately.

### Clinical outcomes and follow-up

For safety reasons, all patients were scheduled for myocardial perfusion scintigraphy (MPS) at 6 ± 2 weeks after discharge to assess residual ischemia. All MPS examinations were analyzed at a core laboratory, with the investigator (A.D.K.) blinded to treatment status. Scans were “nested” (used for study purposes only) but, for safety reasons, made available to physicians if the ischemic burden was >20%.

Telephone follow-up was conducted at 6 months for adverse clinical events. Patients were seen at 9 to 12 months to document major adverse cardiac events (MACE) comprising all-cause mortality, recurrent MI, heart failure (HF), and ischemic-driven revascularization by PCI/coronary artery bypass grafting (CABG). Pre-specified secondary endpoints included cardiovascular death, individual components of the primary endpoint, and the safety endpoints of stroke, major bleeding, and contrast-induced nephropathy.

Clinicians blinded to the randomization group adjudicated all MACE and safety endpoints (definitions provided in Online Appendix 2). Ethics Committee permission was given to approach patients who were randomized in the study but who did not consent to continued participation, to verify vital status at 12 months.

#### Recommended follow-up for symptoms

Trial protocol recommended that chest pain symptoms be primarily evaluated by noninvasive imaging for myocardial ischemia, before angiography with or without PCI. PCI was allowed for ongoing Canadian Cardiovascular Society class III symptoms (despite the use of 2 antianginal medications at maximum tolerated doses) with a negative ischemia test, or if the patient had been admitted with further acute coronary syndrome (ACS). If a patient developed symptoms within 1 month of the 6-week “trial MPS,” this could be revealed to the supervising physician. If more than 1 month had passed since trial MPS, then repeat noninvasive ischemia testing was recommended.

#### Trial governance

The sponsor was the University Hospitals of Leicester National Health Service (NHS) Trust. The Trial Steering Committee was chaired by an independent cardiovascular physician (H.S.) and included 2 independent clinical members and representatives of the lay public, the sponsor, and the funder. An Independent Data Safety and Monitoring Board met regularly and recommended that the trial continue to completion. The independent Clinical Trials and Evaluation Unit at the Royal Brompton & Harefield NHS Foundation Trust (part of the National Institute for Health Research–registered Imperial Clinical Trials Unit) managed the data.

### Statistical analysis

The primary outcome variable, comprising 4 outcomes, was analyzed by time-to–first event survival analysis (log-rank test). The primary analysis was on an intention-to-treat basis of all randomized patients according to treatment group. Kaplan-Meier curves were plotted for randomization to the occurrence of the clinical outcomes and compared by use of the log-rank test, and Cox proportional hazard models were fitted to estimate hazard ratios and 95% confidence intervals for treatment comparisons. The sample size calculation was based on an estimated primary efficacy endpoint of average 12-month MACE, from previous randomized trials, including 12-month cutoff data from the study by Politi et al. 6, 9, 10. On the basis of these figures (average 37% MACE in the IRA-only PCI group vs. 22% in the complete revascularization group), for α level 0.05 and 80% power, the calculated sample size was 144 patients per group. There were 3 pre-specified subgroup analyses: multivessel disease (2 vs. 3), sex, and age (≤65 or >65 years). Continuous data were examined for normality and expressed as mean ± SD or median (IQR) and compared with the Student *t* test or Wilcoxon test as appropriate. Binary event outcomes were expressed as number (%) of patients, and comparisons were performed with the chi-square or Fisher exact test. Two statisticians independently analyzed all data.

Four of the authors (W.M., D.W., M.F., and A.H.G.) had full access to the data (after data lock), and all authors and the trial Steering Committee agreed with the decision to submit the paper. The funder did not play any role in data collection, analysis, interpretation, writing of the manuscript, or the decision to submit the paper.

## Results

**Figure 1:**
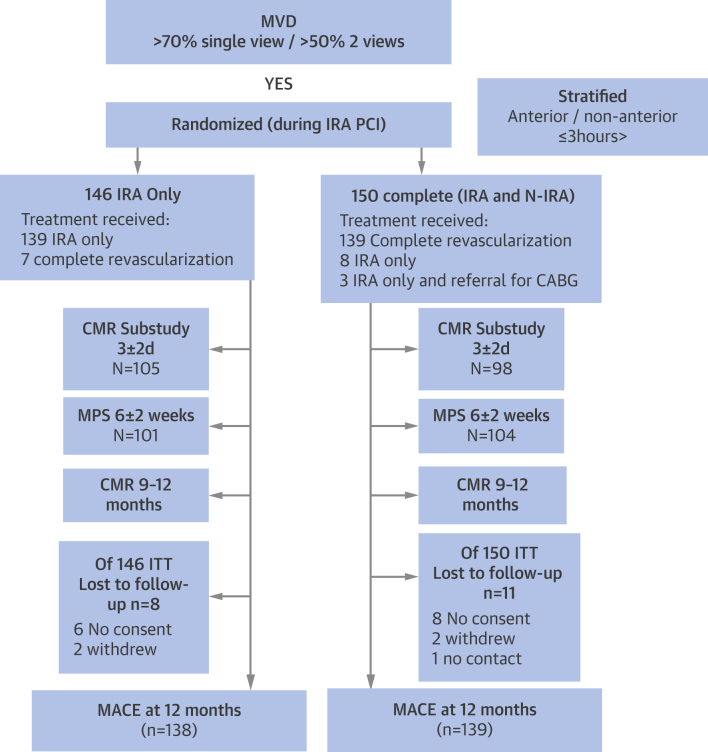
Patient Flow Diagram CONSORT (Consolidated Standards of Reporting Trials) diagram of recruitment to the CvLPRIT study. From 850 patients with ST-segment elevation myocardial infarction, 296 were randomized to receive complete (150) or culprit lesion–only (146) revascularization. Randomized patients were followed up for 12 months, and analysis was by intention-to-treat. CABG = coronary artery bypass grafting; CMR = cardiac magnetic resonance; IRA = infarct-related artery; ITT = intention-to-treat; MACE = major adverse cardiovascular event(s); MPS = myocardial perfusion scintigraphy; MVD = multivessel disease; N-IRA = non–infarct-related artery; PCI = percutaneous coronary intervention.

We screened 850 patients for inclusion, and 296 were randomized (146 to IRA only and 150 to complete revascularization) (Figure 1). Patients in each treatment arm were well matched by demographic and baseline clinical characteristics, although there was a nonsignificant trend for more women in the IRA-only group. The proportion with 2- or 3-vessel disease, lesion locations, and N-IRA stenoses >70% were similar in each group (Table 1).

**Table 1:** Demographics and Baseline Clinical Characteristics

	Complete Revascularization (n = 150)	IRA-Only Revascularization (n = 146)	p Value
Age, yrs	64.6 ± 11.2	65.3 ± 11.9	0.57
Male	128 (85.3)	112 (76.7)	0.06
Treated diabetes	19/147 (12.9)	20/140 (14.3)	0.74
Treated hypertension	54/147 (36.6)	51/140 (36.4)	0.96
Treated hypercholesterolemia	41/147 (27.9)	34/140 (24.3)	0.49
Current smoker	50/146 (34.3)	37/138 (26.8)	0.17
Previous MI	7/147 (4.8)	5/140 (3.6)	0.62
Previous PCI	6/147 (4.1)	3/140 (2.1)	0.50
Killip class II/III on admission	10/147 (6.8)	13/139 (9.4)	0.43
GFR <30 ml/min	1/140 (0.7)	1/137 (0.7)	1.00
Anterior MI	54/150 (36.0)	52/146 (35.6)	0.94
IRA site (selected CASS)			
1 Proximal RCA	29 (19.3)	30 (20.5)	
2 Mid RCA	23 (15.3)	24 (16.4)	0.82
11 LMS	0	0	
12 Proximal LAD	29 (19.3)	31 (21.2)	
13 Mid LAD	22 (14.7)	16 (11.0)	
18 Proximal Cx	9 (6.0)	13 (8.9)	
Other	38 (25.3)	32 (21.9)	
N-IRA anatomic site (selected CASS)			
1 Proximal RCA	23 (15.3)	22 (15.1)	
2 Mid RCA	24 (16.0)	23 (15.8)	0.96
11 LMS	1 (0.7)	2 (1.4)	
12 Proximal LAD	27 (18.0)	21 (14.4)	
13 Mid LAD	44 (29.3)	49 (33.6)	
18 Proximal Cx	20 (13.3)	20 (13.7)	
Other	11 (7.3)	9 (6.2)	
N-IRA stenoses >70%	131 (87.3)	118 (80.8)	0.12
2-Vessel disease	119 (79.3)	110 (75.3)	
3-Vessel disease	31 (20.7)	36 (24.7)	0.41
Symptom to balloon time, min	182 (115-282)	159 (119-265)	0.41
Maximum HS-TnT elevation	985 (629-1,625)	1073 (509-1,824)	0.96
EF (by CMR), %	45.8 ± 9.8 (n = 100)	45.1 ± 9.5 (n = 103)	0.57
Balloon pump	2 (1)	1 (0.6)	1.00
Radial approach	112/146 (76.7)	102/140 (72.9)	0.45

Values are mean ± SD, n (%), n/N (%), or median (interquartile range).

CASS = Coronary Artery Scoring System; CMR = cardiac magnetic resonance; Cx = circumflex; EF = ejection fraction; GFR = glomerular filtration rate; HS-TnT = high-sensitivity troponin T; IQR = interquartile range; IRA = infarct-related artery; LAD = left anterior descending; LMS = left main stem; MI = myocardial infarction; N-IRA = noninfarct-related artery; PCI = percutaneous coronary intervention; RCA = right coronary artery.

In the IRA-only arm, 7 patients (5%) crossed over to receive complete revascularization (Figure 1). In the complete revascularization group, 11 patients (7%) received IRA PCI only, with 3 of these referred for CABG. On the basis of the operator’s clinically driven decision, 64% of the complete revascularization group received N-IRA revascularization at the same procedural session as IRA P-PCI.

Periprocedural details and discharge medications are shown in Table 2. Although the groups were well matched, there was increased usage of thrombectomy catheters in the IRA-only arm. DES use was high in both groups. The total number of stents implanted per patient, procedure time, and contrast volume load were significantly higher in the complete revascularization group. Discharge medication was similar between the 2 groups (Table 2), with very high use of standard optimal medical therapy. Median length of stay was similar between the groups: 3 days (IQR: 2 to 4 days; p = 0.828).

**Table 2:** Periprocedural Details, Discharge Medication, and Ischemia Testing

	Complete Revascularization (n = 150)	IRA-Only Revascularization (n = 146)	p Value
ASA	141/142 (99.3)	131/135 (97.0)	0.16
Plus clopidogrel	59/144 (41.0)	54/138 (39.1)	0.75
Plus ticagrelor	19/144 (13.2)	18/135 (13.3)	0.97
Plus prasugrel	58/144 (40.3)	64/138 (46.4)	0.30
Plus warfarin	1/147 (0.7)	2/138 (1.5)	0.61
GPI	46/145 (31.7)	44/139 (31.7)	0.99
Bivalirudin	79/139 (56.8)	65/128 (50.8)	0.32
TIMI flow grade 0/1 on arrival	120/147 (81.6)	118/140 (84.3)	0.55
Thrombus aspiration catheter used	93/145 (64.1)	105/140 (75.0)	0.047
DES	141/147 (95.9)	127/140 (90.7)	0.08
Stents per patient	3 (2–4)	1 (1–2)	<0.0001
Total procedure time, min	55 (38–74)	41 (30–55.5)	<0.0001
Total contrast used, ml	250 (190–330)	190 (150–250)	<0.0001
Beta-blocker	137/147 (93.2)	126/135 (93.3)	0.96
ACEI/ARB	142/147 (96.6)	129/135 (95.6)	0.65
Statin	146/146 (100)	133/135 (98.5)	0.14
Aldosterone antagonist	9/147 (6.1)	8/135 (5.9)	0.95
Other antianginal agent	55/147 (37.4)	49/135 (36.3)	0.85
Loop diuretic agent	15/147 (10.2)	17/135 (12.6)	0.53

Values are n/N (%) or median (interquartile range).

ACEI/ARB = angiotensin-converting enzyme inhibitor/angiotensin receptor blocker; ASA = acetylsalicylic acid; DES = drug-eluting stent(s); GPI = glycoprotein IIb/IIIa inhibitor; IRA = infarct-related artery; TIMI = Thrombolysis In Myocardial Infarction.

### Ischemia testing

As a safety measure to detect high residual ischemic burden, 205 patients agreed to undergo MPS, of whom 203 completed the rest/stress protocol (100 from the IRA-only group and 103 from the complete revascularization group), at a median of 7 weeks (IQR: 6.3 to 8.6 weeks) in the complete group and 6.9 weeks (IQR: 6.3 to 8.3 weeks) in the IRA-only group. No scans required protocol-mandated unblinding as a result of ischemic burden >20%. A full analysis of the MPS data is under way.

### Clinical outcomes

Among those patients randomized after verbal assent (Central Illustration), the median follow-up was 364 (IQR: 286 to 365) days. Nineteen patients were lost to follow-up (Figure 1). These patients were tracked through a U.K. national database for their vital status, which confirmed that none had died.

**Central Illustration:**
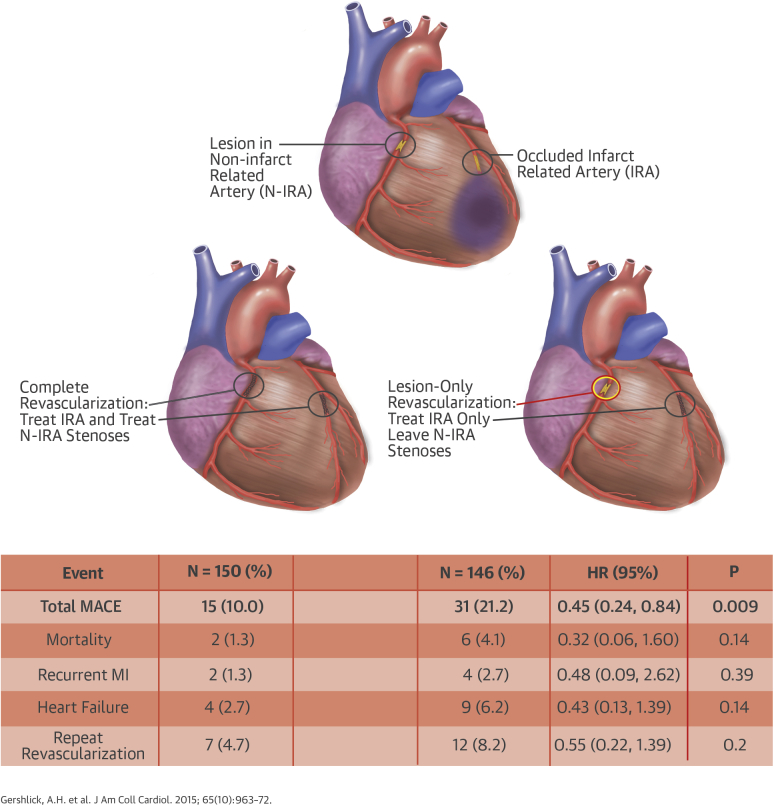
Complete Versus Lesion-Only Revascularization in Acute MI Overview of the CvLPRIT trial showing the randomization strategy and main results. CI = confidence interval; CvLPRIT = Complete Versus Lesion-Only Primary PCI trial; HR = hazard ratio; IRA = infarct-related artery; MACE = major adverse cardiac event(s); MI = myocardial infarction; N-IRA = non–infarct-related artery.

**Figure 2:**
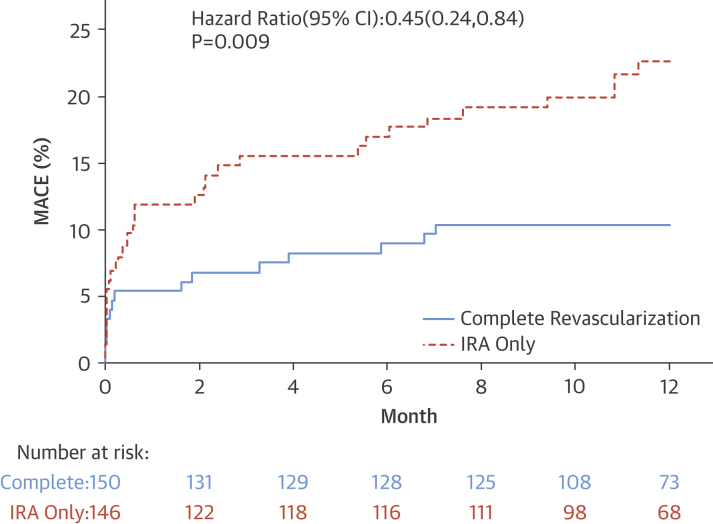
Kaplan-Meier Curves Cumulative event rate for IRA-only versus complete revascularization groups. CI = confidence interval; other abbreviations as in Figure 1.

The primary endpoint is presented as time to first event (Table 3). MACE was significantly lower in the complete revascularization arm (10.0%) than in the IRA-only arm (21.2%; hazard ratio: 0.45; 95% confidence interval: 0.24 to 0.84; p = 0.009). The individual components of the primary endpoint and cardiovascular mortality were all also lower, although none were statistically significant. The Kaplan-Meier curves (Figure 2) showed early divergence, with continuing separation during follow-up. The forest plot for pre-specified subanalyses is shown in Online Figure 1. Kaplan-Meier curves to 30 days are shown in Online Figure 2.

**Table 3:** Clinical Outcomes at 12 Months

	Complete Revascularization (n = 150)	IRA-Only Revascularization (n = 146)	HR (95% CI)	p Value
Time to first event				
MACE	15 (10.0)	31 (21.2)	0.45 (0.24–0.84)	0.009
All-cause mortality	2 (1.3)	6 (4.1)	0.32 (0.06–1.60)	0.14
Recurrent MI	2 (1.3)	4 (2.7)	0.48 (0.09–2.62)	0.39
HF∗	4 (2.7)	9 (6.2)	0.43 (0.13–1.39)	0.14
Repeat revascularization	7 (4.7)	12 (8.2)	0.55 (0.22–1.39)	0.20
All events				
All-cause mortality	4 (2.7)	10 (6.9)	0.38 (0.12–1.20)	0.09
Recurrent MI	2 (1.3)	4 (2.7)	0.47 (0.09–2.59)	0.38
Type 1	0	2		
Type 4b	2	2		
HF	5 (3.3)	10 (6.9)	0.47 (0.16–1.38)	0.16
Inpatient	3	7		0.56
Post-discharge	2	3		
Repeat revascularization	8 (5.3)	16 (11.0)	0.46 (0.20–1.08)	0.07
Safety				
CV mortality	2 (1.3)	7 (4.8)	0.27 (0.06–1.32)	0.11
Stroke	2 (1.3)	2 (1.4)	0.95 (0.13–6.77)	0.96
Major bleed	4 (2.7)	7 (4.8)	0.55 (0.16–1.87)	0.34
Contrast-induced nephropathy	2 (1.4)	2 (1.4)	0.94 (0.13-6.75)	0.95

Values are n (%) for occurrences of both first events and total events. Outcomes are shown according to MACE and individual components of MACE (death, MI, HF, and revascularization) at 12 months.

CI = confidence interval; CV = cardiovascular; HF = heart failure; HR = hazard ratio; MACE = major adverse cardiac event; other abbreviations as in Table 1.

∗ Of the 13 patients with failure events, 5 (4 in the IRA-only group and 1 in the complete revascularization group) subsequently died during the 12-month follow-up.

There was no increase in stroke, major bleeding (all non-CABG related), or contrast-induced nephropathy in the complete revascularization group (Table 3). Outcomes were similar between intention-to-treat groups, per protocol (Online Table 2), and in the as-treated population (Online Table 3). There was a significant reduction in the exploratory combined endpoint of all-cause mortality, recurrent MI, or HF in the complete revascularization group (p = 0.025) (Online Table 4). There was a trend to reduced MACE in the population undergoing complete revascularization at the same sitting as IRA P-PCI compared with those having a staged procedure (Online Table 5).

### Subsequent revascularization

Reasons for revascularization are shown in the following text (excluding patients who underwent revascularization after presentation with acute MI, which were classified hierarchically as recurrent MI).

#### Complete revascularization group (7 events)

One repeat PCI was symptom driven, 1 followed admission with troponin-negative ACS, 1 patient underwent attempted repeat PCI after failed N-IRA PCI at initial P-PCI, 3 underwent CABG after P-PCI to the IRA only at index admission (2 as outpatients, 1 as urgent inpatient CABG day 3 after P-PCI); and 1 patient had IRA-only revascularization and was readmitted electively at 6 weeks for further revascularization to the N-IRA.

#### IRA-only revascularization group (12 events)

Ten patients underwent PCI for recurrent chest pain (1 with positive MPS, 5 admitted with troponin-negative ACS, and a further 4 on the basis of clinician judgment after optimal [>2] antianginal therapy). One patient underwent elective N-IRA PCI after discharge at the operator’s discretion, and 1 underwent CABG for refractory angina.

## Discussion

CvLPRIT has demonstrated that in patients undergoing P-PCI for STEMI, complete revascularization during the index admission resulted in a significantly lower MACE rate at 12 months than when only the IRA was treated, with early separation of clinical event curves. Although there were numerically fewer events in individual components of the composite primary endpoint in the complete revascularization group, the trial was not powered to detect significant differences in MI or death. After the exclusion of revascularization, there was a significant reduction in the exploratory combined endpoint of all-cause mortality, recurrent MI, or HF in the complete revascularization group.

A recent meta-analysis has confirmed that multivessel disease is commonly present among STEMI patients presenting for P-PCI and has a negative impact on 30-day mortality (11). However, previous studies addressing the management of N-IRA lesions have produced conflicting results, as reflected in the 2013 American College of Cardiology Foundation/American Heart Association (12) and 2012 European Society of Cardiology (13) guidelines (IIIC and IIb Class C, respectively). These recommendations were made on the basis of studies that differed in design and consisted of subgroups of randomized P-PCI trials (2) or retrospective observational registries (4) and that suggested that in-hospital complete revascularization appeared to be associated with worse outcome. The reason for a trend toward increased mortality with immediate complete PCI in the nonrandomized retrospective registry studies is likely attributable to case selection. As acknowledged by the authors of the largest published registry from New York, retrospective studies are intrinsically susceptible to selection bias, despite attempts to mitigate this by propensity matching (4). In that registry, multivessel PCI was performed in only 12.5% of the 4,024 STEMI patients with multivessel disease. Complete PCI in nonrandomized studies may define a sicker cohort of STEMI patients who die of their underlying disease rather than of a particular treatment strategy. There are few randomized data that assess the outcomes of complete revascularization after STEMI in equitable patient populations. Recently, however, 1 randomized trial, PRAMI (5), reported clear clinical benefit in treating both the IRA and N-IRA at the index procedure. However, this single trial has not resulted in a widespread change in clinical practice, which makes the positive results of CvLPRIT particularly relevant, not least because a review of the data after trial presentation led to a change in the American College of Cardiology/American Heart Association recommendation (14).

CvLPRIT data, in particular, showed early separation of the MACE curves, which in itself warrants discussion. In general, treatment of a significant stenosis by PCI, especially in stable patients, has been thought to lead to symptom improvement, rather than to influence prognosis. The recently published network meta-analysis by the European Myocardial Revascularization Collaboration suggests a potential mortality benefit as well (15). This intriguing result calls our understanding of clinically silent “bystander” disease into question. There is emerging evidence of pan-coronary inflammation (16) and the presence of multiple unstable coronary plaques (17), which may explain the higher incidence of recurrent ACS after STEMI than in patients with stable coronary disease (18). Pacification of these bystander plaques may be proposed as a mechanism of benefit in complete revascularization. However, in our relatively small study, there were only 2 fewer spontaneous MIs (type 1) in the complete revascularization group than in the IRA-only group, which suggests that any effect of plaque pacification may be relatively small, but this should be the focus of mechanistic understanding in future larger trials.

The role of fractional flow reserve (FFR) in assessing the importance of the N-IRA in STEMI patients is contentious and is being compared in a number of ongoing studies with angiographically guided intervention. In the original FFR study by Dambrink et al. (19), 40% of nonculprit lesions did not show hemodynamic significance (FFR >0.75). In the same group’s follow-up study (20), FFR-guided intervention resulted in no difference in 3-year MACE. Although FFR was shown to be safe, its incremental value over angiographic severity (as used in CvLPRIT) to pragmatically reflect current clinical practice is as yet unproven. Future studies may address the specific value of instantaneous FFR.

There was a nonsignificant trend toward reduced HF events with complete revascularization. Additionally, there was an incidence of pre-discharge HF, which prolonged hospitalization, with double the number (n = 7) in the IRA-only group compared with the complete revascularization group (n = 3). Importantly, there were 5 deaths (4 in the complete revascularization group and 1 in the IRA-only revascularization group) in patients with HF as a first event, which confirms the poor prognosis of patients with HF after STEMI. Although these numbers are small, one could speculate that N-IRA treatment works through resolving early myocardial stunning/hibernation. It is well recognized that hibernation that results from severe coronary artery disease can contribute to the development of HF. Multiple studies with different imaging techniques have shown that revascularization of hibernating myocardium results in improved left ventricular function, and a meta-analysis suggests this is associated with improved clinical outcomes (21). As well as preventing hibernation, N-IRA PCI may also improve myocardial salvage by increasing blood flow to watershed areas of infarction, which could translate into improved clinical outcomes.

Importantly, in the context of an open-label study, the primary endpoint was not driven by differences in revascularization rates alone. Specifically, the reductions in the hazard ratio for death/recurrent MI and death/MI/HF were at least as great as that for repeat revascularization and were statistically significant for death/MI/HF (Table 3). We acknowledge, however, that our definition of MI used was strict, because recurrent MI had to be driven by symptoms and was not recorded as being caused by increased periprocedural troponin increases alone (22). Because of the large enzyme release from the STEMI, small periprocedural infarcts related to revascularization of the non-IRA may have been missed. The frequency and size of these potentially important type 4b infarcts (23), as well as the effect on myocardial salvage and hibernating myocardium, will be addressed in the to-be-published cardiac magnetic resonance substudy. Further insights regarding the effect of complete revascularization on ischemia and how this relates to prognosis will be gathered from a nuclear imaging substudy that will be reported separately.

The subgroup analyses suggested benefit in all subgroups, particularly for women, those older than 65 years of age, and those with double-vessel disease, although the analyses were limited by the small sample size. Of the 139 patients who had complete revascularization, approximately two-thirds had N-IRA PCI at the same sitting as the P-PCI. Those patients with immediate N-IRA PCI showed a strong trend toward improved clinical outcomes compared with those undergoing a staged in-hospital procedure. No firm conclusions can be drawn, because the numbers in this arm were small; staging was based on clinicians’ decisions and was nonrandomized. However, the early separation of the event curves in CvLPRIT suggests that a delayed staged outpatient complete revascularization strategy may not be as effective as in-hospital treatment.

The high incidence of radial access, DES use, and optimal secondary preventative medication in CvLPRIT make it a contemporary P-PCI trial. The number and severity of treated N-IRA lesions in CvLPRIT suggest that difficult multivessel disease cases were included; a small proportion of screened cases were not considered eligible because of anatomy or operator discretion (47 of 850 patients). There was no increase in any of the secondary safety endpoints, which is reassuring, although further definitive data will be required from larger trials.

Our results are consistent with those seen in the PRAMI trial (5). CvLPRIT and PRAMI were similar in general design; both assessed the safety and benefit of total revascularization intended around the time of P-PCI. There were, however, important methodological differences, which included composite endpoint definitions. In general, the results of CvLPRIT and PRAMI are strikingly similar and add further credence to the outcome data seen in PRAMI. Particular features of CvLPRIT included randomization before IRA PCI was completed and its design, which allowed clinicians to defer the N-IRA procedure from the index P-PCI until later during the index admission, if clinically indicated.

The results of both CvLPRIT and PRAMI provide randomized data that argue that there may well be an advantage to undertaking complete revascularization in STEMI patients. Further large-scale randomized trials to address the impact of complete revascularization on hard endpoints (death/recurrent MI), FFR estimation of N-IRA lesion severity, and the timing of N-IRA treatment (inpatient vs. outpatient) are critically needed and ongoing. CvLPRIT and PRAMI have added to the evidence base regarding that management of multivessel disease at P-PCI and provide an important basis for the robust design of future trials.

### Study limitations

The trial was not powered to show differences in components of the primary endpoint, and larger trials powered for death and MI are needed. Eleven patients in the complete revascularization group and 8 in the IRA-only group were lost to follow-up; however, none of these patients died. Case-selection bias cannot be completely excluded, but because of the very nature of multivessel disease clinical trials in the P-PCI setting, it is not possible to randomize patients before the results of the angiogram are known, and such a design is standard for trials of this type. Repeat revascularization may have been influenced by staff and patient knowledge of untreated other “significant” disease, although fewer hard endpoints were detected in the complete revascularization group. Because this was a pragmatic study, neither intravascular ultrasound nor FFR was used to assess lesion severity. Future trials will address their value. Longer-term follow-up is awaited, although the event curves continue to diverge at 1 year.

## Conclusions

CvLPRIT has demonstrated that in a population of patients with STEMI treated by contemporary P-PCI, in-hospital complete revascularization of angiographically significant N-IRA lesions results in improved clinical outcomes compared with treatment of the culprit lesion only. There was no significant reduction in death or MI, and larger clinical trials are needed to address these specific endpoints.

Perspectives **COMPETENCY IN MEDICAL KNOWLEDGE:** In 2 randomized trials reported to date, percutaneous interventions on non–infarct-related stenotic coronary arteries reduced MACE, which led to withdrawal of class III practice guideline recommendations not to treat significant non–infarct-related stenoses in patients with STEMI and multivessel disease undergoing primary angioplasty.

**TRANSLATIONAL OUTLOOK:** More clinical trials are needed to verify whether complete revascularization prevents reinfarction and mortality, define the optimal timing of multivessel revascularization, and determine whether interventional decisions guided by functional assessment of coronary lesions improve outcomes.
