# Supplementary material for: Randomized Trial of Complete Versus Lesion-Only Revascularization in Patients Undergoing Primary Percutaneous Coronary Intervention for STEMI and Multivessel Disease: The CvLPRIT Trial
Source: J Am Coll Cardiol. 2015 Mar 17;65(10):963–72. doi: 10.1016/j.jacc.2014.12.038 (PMC4359051; doi:10.1016/j.jacc.2014.12.038)
Supplement: Online Data [file mmc1.docx]

**Supplementary Table 1: Inclusion and Exclusion criteria**

| **Inclusion criteria**  Suspected or proven acute myocardial infarction; Significant ST elevation or left bundle branch block (LBBB) on ECG (in cases of LBBB, angiographic confirmation of IRA occlusion is required)  < 12 hrs of symptom onset  Scheduled for Primary PCI for clinical reasons  Provision of verbal assent followed by written informed consent  Multivessel coronary artery disease at angiography defined as:  Infarct related artery (IRA) plus at least one non-infarct related epicardial artery (N-IRA) with at least one lesion deemed angiographically significant (>70% diameter stenosis in one plane or > 50% in 2 planes).  The N-IRA should be a major (>2mm) epicardial coronary artery or branch (>2mm) and be suitable for stent implantation. |
| --- |
| **Exclusion criteria**  Any exclusion criteria for P-PCI  <18 years  Clear indication for, or contraindication to, multi vessel P-PCI according to operator judgement  Previous Q wave myocardial infarction  Patients with prior CABG  Cardiogenic Shock  VSD or moderate/severe mitral regurgitation  Chronic kidney disease (Cr>200μmol/l or eGFR<30ml/min/1.73m^2^)  Suspected or confirmed thrombosis of a previously stented artery  Where the only significant N-IRA lesion is a chronic total occlusion |

**Supplementary Table 2: Clinical Outcomes at 12 Months in the Per Protocol Population**

| Variable | Complete Revascularization | IRA only  Revascularization | HR (95% CI) | P |
| --- | --- | --- | --- | --- |
| MACE | 9/136 (6.6) | 28/138 (20.3) | 0.31 (0.15, 0.65) | 0.0011 |
|  |  |  |  |  |
| All-cause mortality | 1/136 (0.7) | 5/138 (3.6) | 0.20 (0.02, 1.73) | 0.106 |
| Recurrent MI | 2/136 (1.5) | 4/138 (2.9) | 0.50 (0.09, 2.74) | 0.418 |
| Heart failure | 3/136 (2.2) | 7/138 (5.1) | 0.43 (0.11, 1.66) | 0.207 |
| Repeat Revascularization | 3/136 (2.2) | 12/138 (8.7) | 0.24 (0.07, 0.85) | 0.016 |
|  |  |  |  |  |
| CV mortality | 1/136 (0.7) | 3/138 (2.2) | 0.33 (0.03, 3.22) | 0.343 |
| Stroke | 1/136 (0.7) | 2/138 (1.4) | 0.50 (0.04, 5.47) | 0.559 |
| Major Bleed | 3/136 (2.2) | 6/138 (4.3) | 0.50 (0.12, 1.99) | 0.314 |

MACE= Major Adverse Cardiac Event (all-cause mortality, recurrent myocardial infarction, heart failure hospital admission, repeat revascularisation). Results are shown as number of patients per group with event (%). The numbers in each treatment are equal to the number who received the treatment they were assigned to at randomisation and did not have any protocol violation (n=4)

**Supplementary Table 3.**

**Clinical Outcomes in the ‘As-Treated’ Population**

| Variable | Complete Revascularization  (N=146) | IRA only Revascularization (N=147) | HR (95% CI) | P value |
| --- | --- | --- | --- | --- |
| MACE | 13 (8.9) | 30 (20.4) | 0.42 (0.22,0.80) | 0.0062 |
| All-cause mortality | 3(2.1) | 5(3.4) | 0.60 (0.14,2.51) | 0.48 |
| Recurrent MI | 2 (1.4) | 4 (2.7) | 0.50 (0.09,2.71) | 0.41 |
| Heart failure | 5(3.4) | 8(5.4) | 0.63(0.20,1.91) | 0.41 |
| Repeat Revascularization | 3 (2.1) | 13 (8.8) | 0.22 (0.06,0.76) | 0.0086 |

Note: 3 patients in the complete revascularization arm who had IRA-only and were referred for CABG are not included in this analysis. Results are shown as number of patients per group with event (%).MACE= Major Adverse Cardiac Event (all-cause mortality, recurrent myocardial infarction, heart failure hospital admission, repeat revascularisation)

**Supplementary Table 4: Clinical Outcomes at 12 Months Excluding Repeat Revascularization in the ITT Population**

| **Variable** | **Complete Revascularization** | **IRA only** | **HR (95% CI)** | **P** Value |
| --- | --- | --- | --- | --- |
| All-cause mortality or Recurrent MI | 6/150 (4.0) | 14/146 (9.6) | 0.41 (0.16, 1.07) | 0.060 |
| All-cause mortality, Recurrent MI or Heart Failure | 8/150 (4.7) | 19/146 (13.0) | 0.40 (0.18, 0.92) | 0.025 |

ITT = intention to treat. Data analyzed on time to first event. HR= hazard ratio CI= Confidence interval. Results are shown as number of patients per group with event (%).

**Supplementary Table 5: Clinical Outcomes at 12 Months: Immediate Versus Delayed (Staged) Complete Revascularization**

| Clinical Outcome | No (%) of patients with event | | HR (95% CI) | p |
| --- | --- | --- | --- | --- |
|  | Same Sitting (97) | Delayed (42) |  |  |
| MACE (Death/MI/HF/Revascularization) | 6 (6.2) | 5 (11.9) | 0.51 (0.16, 1.67) | 0.27 |
| MACE (Death/MI/HF) | 3 (3.1) | 5 (11.9) | 0.26 (0.06, 1.08) | 0.06 |

N.B These data are for those patients (n=139) who received complete revascularization as intention to treat (see Figure 1). Results are shown as number of patients per group with event (%).MACE= Major Adverse Cardiac Event (all-cause mortality, recurrent myocardial infarction, heart failure hospital admission, repeat revascularization)

**Appendix 1 Author affiliations**

1. Department of Cardiovascular sciences, University of Leicester and the NIHR Leicester Cardiovascular Biomedical Research Unit, University Hospitals of Leicester NHS Trust, Glenfield Hospital, Leicester, LE3 9QP, UK
2. Royal Derby Hospital, Derby DE22 3NE, UK
3. Multidisciplinary Cardiovascular Research Centre & The Division of Cardiovascular and Diabetes Research, Leeds Institute of Cardiovascular and Metabolic Medicine (LICAMM), University of Leeds, Leeds, LS2 9JT, UK
4. Clinical Trials & Evaluation Unit, Royal Brompton & Harefield NHS Foundation Trust and Imperial Clinical Trials Unit, Imperial College London, London, UK
5. University Hospital Southampton NHS Foundation Trust, Faculty of Medicine, University of Southampton, Southampton SO16 6YD,UK
6. Department of Cardiology, Leeds Teaching Hospitals NHS Trust, Leeds, LS1 3EX, UK
7. Royal Brompton and Harefield NHS Trust, Sydney Street, London SW3 6NP, UK
8. Clinical Research Network: East Midlands, Leicester Royal Infirmary, LE1 5WW, UK
9. Cardiovascular Biomedical Research Unit, Royal Brompton and Harefield NHS Trust, Sydney Street, London, SW3 6NP, UK
10. Department of Clinical Sciences, Liverpool School of Tropical Medicine, Liverpool, L3 5QA, United Kingdom
11. Norfolk and Norwich University Hospitals NHS Foundation Trust and Norwich Medical School, University of East Anglia Norwich NR4 7TJ, UK
12. Kettering General Hospital, Rothwell Road, Kettering NN16 8UZ, UK
13. Oxford Heart Centre, John Radcliffe Hospital,OxfordOX3 9DU, UK
14. Royal Bournemouth Hospital, Castle Lane East, Bournemouth BH7 7DW, UK
15. North Staffordshire Hospital Clayton Road Newcastle-under-Lyme, Staffordshire ST5 4DB
16. The Heart Hospital, University College London Hospitals, 16-18 Westmoreland Street, London W1G 8PH, UK

**Appendix 2 Definitions outcome clinical events**.

**1. Death**

Death from any cause classified as cardiovascular or non-cardiovascular. Cardiovascular death includes any cardiac causes, or other vascular causes (e.g. pulmonary embolism, aortic dissection).

**2. Myocardial infarction (MI):**

Troponin level is not routinely measured post PCI. Myocardial infarction will require a hospital admission, or be diagnosed in hospital, with one or more of the following:”: **Type 1:** Spontaneous re-MI: Recurrent angina symptoms or new ECG changes occurring before PCI or <48 hours from PCI that is compatible with re-MI associated with an elevation of CK-MB, troponin, or total CK beyond ULN and 20% or more above the previous value. **Type 4a:** CK-MB or total CK >3 times the ULN within 48 hours following PCI. If the pre-PCI CK-MB or total CK level is higher than the ULN, there also needs to be:

either the demonstration of a falling CK-MB or total CK level prior to the onset of the suspected event,

or a subsequent peak of the cardiac biomarker of at least 20% above the previous value obtained prior to the onset of the suspected event.

With either an appropriate clinical presentation or new ischemic ECG changes (ST-segment depression or ST-segment elevation or development of new pathological Q waves/LBBB).

Type 4b: Myocardial infarction associated with stent thrombosis as documented by angiography or at autopsy AND fulfilling the criteria of spontaneous MI (Type 1)

**3. Heart failure**

Heart failure will be defined as a hospital admission with any of the following symptoms and signs: worsening breathlessness, fatigue, fluid overload, pulmonary edema, elevated venous pressure and elevated BNP. Confirmation of heart failure according to local expert judgment and evidence of impaired LV function will be required for the event to be classified as heart failure.

**4. Repeat Revascularization**

Repeat revascularizations classified as:

1. Target lesion re-interventions (TLR) inside the implanted stent or within 5 mm proximally or distally or repeated interventions in the same vessel (TVR) by percutaneous coronary interventions (PCI) or by coronary artery bypass graft surgery.

2. PCI to lesions not identified previously

3. CABG for new symptoms or complications of PCI

**5. Stent thrombosis**

Stent thrombosis (ST) will be classified as “acute”- within 24 hours from the procedure, “sub-acute” up to 30 days, “late” from 30 days till 1 year and “very late” after 1 year after index procedure. Thrombosis will be classified as definite, probable and possible according to the definition of Academic Research Consortium [25]. ST will be defined as the occurrence of one of the following events: 1. Angiographic documentation of complete or partial stent occlusion and target vessel related acute clinical ischemic event. 2. Autopsy documentation of complete or partial thrombotic stent occlusion 3. Myocardial infarction in the distribution of the stented vessel.

We will separately evaluate the incidence of possible ST by including all unexplained death after 30 days.

**6. Emergency CABG**

This will be defined as CABG occurring within 48 hours of an ischemic event in a patient who was not previously scheduled to have CABG.

**7. Stroke**

Defined as the presence of a new focal neurologic deficit thought to be vascular in origin, with signs or symptoms lasting more than 24 hours. It is strongly recommended (but not required) that an imaging procedure such as CT scan or MRI be performed. Stroke will be further classified as ischemic, hemorrhagic or type uncertain.

**8. Major bleeding**

Major bleeding defined as the cumulative occurrence of intracranial or intraocular bleeding, hemorrhage at the vascular access site requiring intervention, a reduction in hemoglobin levels of at least 5 grams per deciliter, reoperation for bleeding or transfusion of a blood product (at least 2 units), bleeding causing substantial hypotension requiring the use of inotropic agents. All other bleeding events were considered as minor (i.e. epistaxis, blood traces in the stool etc.)

**9. Surgical repair of a vascular complication**

In general this will refer to surgical repair to the femoral or radial arteries following P-PCI but could refer to venous complications or in unusual circumstances repairs to the aorta or carotid arteries.

**Appendix 3**

The committee members and investigational team for CvLPRIT trial were as follows:

**Steering Committee**: H. Swanton (Independent Chair), P. Schofield (Independent member), M. Gunning (independent member), A. H. Gershlick, N. Curzen, D. Blackman, J. Greenwood, M. Dalby, G. McCann, A. Kelion, D. Kelly, S. Hetherington, S. Talwar, M. Flather, T. Sasikaran, D. Hetmanski, K. Fairbrother, S. Amoils (BHF), G. Thompson (Lay member).

**Data Safety and Monitoring Board**: R. Hall (Chair), T. Gilbert, M. Roughton

**Event adjudicators:** A.H. Gershlick, M. Gunning, S. Hetherington

**Coordinating Centre:** T.Sasikaran, M. Yanez-Lopez, W. Aslam, D. Babalis, E. Matesanz, E. Zbrzeska, N. Lago, J. Booth, F. Nugara,

**Glenfield Hospital, Leicester: (**Nurses): Lorraine Shipley, Kathryn Fairbrother, Gemma Turland, Emma Parker, Joanna Hughes, Victoria Meynell, Amanda Swinnerton. (Interventional Cardiologists ): Ian Hudson, Elved Roberts, David Adlam, Doug Skehan, NileshSumani, Jan Kovac, Gail Richardson, Raj Rajendra, Albert Alahmar. (Others): Jamal Khan, Sheraz Nazir, David Monk, Mini Pakal, Anna-Marie Marsh, John McAdam

**Harefield Hospital:** (Nurse): Paula Rogers. (Interventional cardiologists): Charles Ilsley,

Rebecca Lane, Piers Clifford, Tito Kabir, Robert Smith. (Other): WalaMattar

**Kettering General Hospital: (**Nurses): Charmaine Beirnes, Amanda Chapman, Howard Fairey,

Michelle Bilson. (Interventional cardiologists): Kai Hogrefe, Martin Sluka, Mohsin Farooq, Naeem Shaukat, Javed Ehtisham, Salman Nishtar.

**Leeds General Infirmary: (**Nurses): Kathryn Somers, Michelle Anderson, Charlotte Harland, Natalie Burton-Wood. (Interventional Cardiologists): C Malkin, JM Blaxill, SB Wheatcroft, UM Sivananthan

**Royal Bournemouth Hospital:** (Nurses) Sarah Orr, Nicki Lakeman

**Royal Derby Hospital: (**Nurses): Fiona Robertson, Marie Appleby, Carmen Lisbey. (Interventional Cardiologists): Tariq Azeem, Julia Baron, Manoj Bhandari, Kamal Chitkara, Alastair McCance. (Others): Jacqui McCance, Anne Bebbington, Teresa Grieve, Richard Donnelly.

**Southampton General Hospital: (**Nurse): Zoe Nicholas. (Interventional Cardiologists): Huon Gray, Iain Simpson, Alison Calver, Simon Corbett, James Wilkinson.


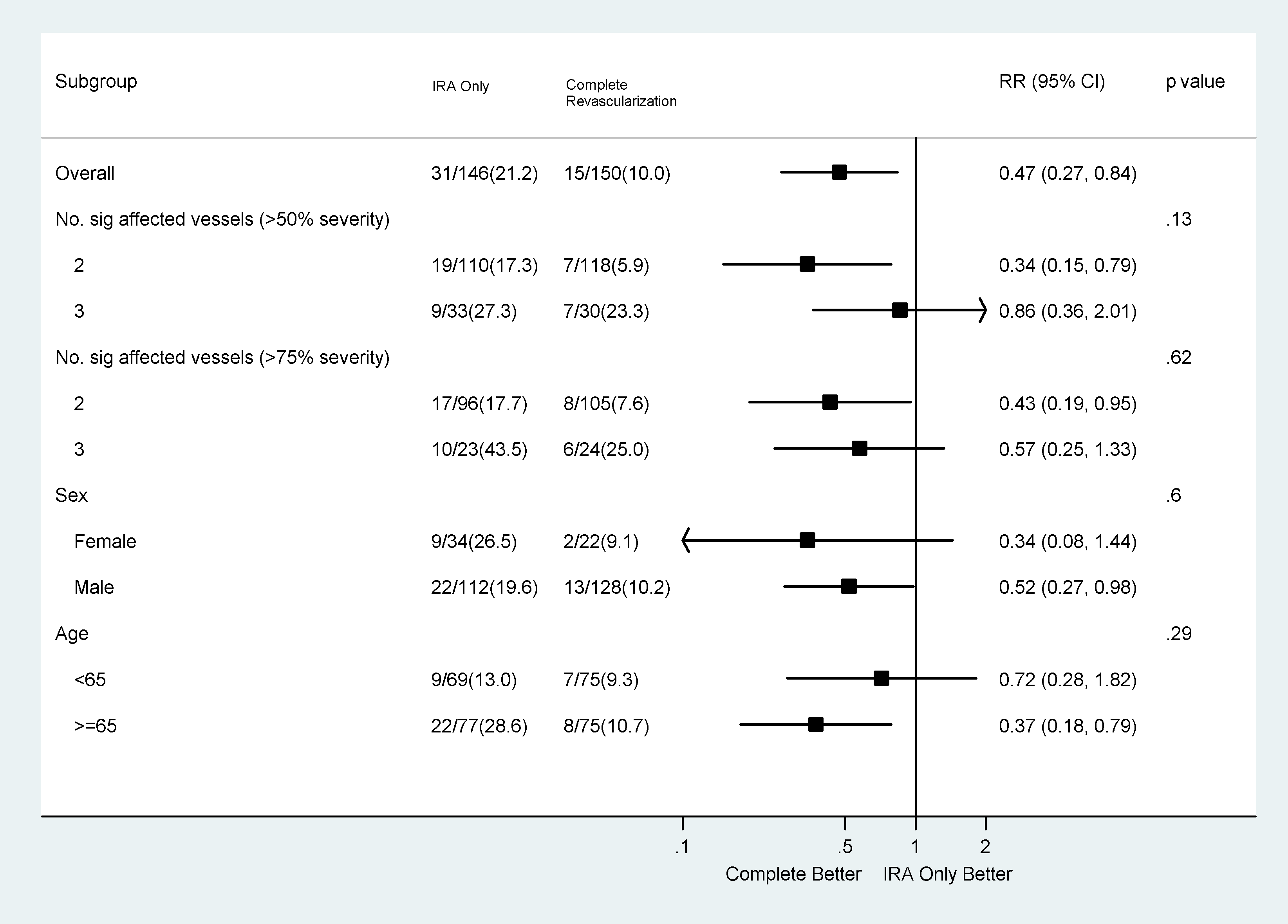


Online Figure 1


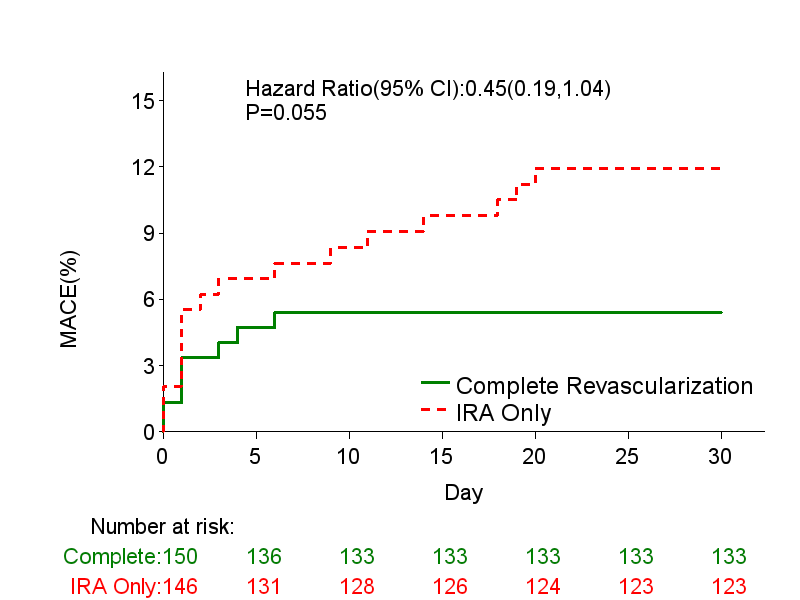


Online Figure 2
